# In Search of Environmental Factors Associated With Global Differences in Birth Weight and BMI

**DOI:** 10.1002/ajhb.70038

**Published:** 2025-04-07

**Authors:** Per M. Jensen, Marten Sørensen

**Affiliations:** ^1^ Department of Plant and Environmental Sciences University of Copenhagen Frederiksberg Denmark

**Keywords:** birth weight, BMI, epidemiological transition, global pattern, mortality, temperature

## Abstract

**Objective:**

The “fetal origin of adult diseases hypothesis” encompasses the notion that intrauterine growth restriction (IUGR) alters fetal development trajectories. Various neonatal metrics inform IUGR, but not all contributors to IUGR have an impact on development trajectories. Chronic IUGR (twins) and slowly varying IUGR (seasonal) have little to no effect on later life trajectories. Perhaps development trajectories may evolve through other mechanisms, as for example, multiple short‐lived periods of IUGR and repeated stimulation of metabolic pathways.

**Methods:**

Daily temperature variation could deliver a frequent IUGR as pregnant women would experience some degree of placental vasoconstriction during maximum/midday temperatures. We assessed the association with daily temperature amplitudes for globally distributed records of crude fetal growth rates (CFGR) and BMI. Paired birthweight (BW) and gestational age (GA) data permitted analyses of CFGR in 70 countries and subsequent analysis of CFGR for association with daily temperature amplitude, seasonal temperature amplitude, mean annual temperature, calorie intake per day per^−1^ person^−1^, BMI, height, and socioeconomic conditions. Analog analyses were performed for gestational age, calorie intake, BMI, and height.

**Results:**

CFGR and BMI showed a clear association with daily temperature amplitudes, which was not the case for gestational age, calorie intake, and height.

**Conclusion:**

We show that daily temperature amplitudes are associated with both CFGR and BMI. These results permit a wider ecological appreciation of the hypothesis because daily temperature amplitudes inform environmental aridity and food scarcity. We discuss how scarcity, affluence, and the epidemiological environment influence the prevalence of afflictions associated with the fetal origin of adult disease hypothesis.

## 1 Introduction

The “thrifty genotype”/“fetal origin of adult diseases”/“developmental origins of health and disease” hypotheses have, in past decades, been accepted as the principal explanation for an array of health challenges affecting current populations in developed countries (Buklijas and Al‐Gailani 2023). The hypothesis was initially developed to address the rising prevalence of obesity, elevated blood pressure, and diabetes in developed countries in the mid‐20th century. Before, these afflictions were seen as the product of a particular lifestyle (e.g., Harvey 1872). All individuals were equally vulnerable, and the condition could be amended by added physical activity and a change in diet. It was, however, increasingly clear that some individuals and certain families were more vulnerable to the adverse effects of a modern lifestyle. Thus, Neel (1962) proposed that the underlying reason was of genetic origin and suggested that people with diabetes represented “A thrifty” genotype rendered detrimental by “Progress”. He made parallels to sickle‐cell anemia to clarify that a genotype could be advantageous under certain environmental conditions, even though it generally had negative health impacts. The negative impact of “progress” was continued by Barker and Osmond (1986), who argued that the underlying cause for later life health challenges was linked to nutritional deficits during fetal development. Thus, the association with nutritional conditions remained, but the origin was associated with differences in growth, that is, a phenotype. Neel (1999) and others (Prentice et al. 2005) continued their work on thrifty genotypes, while others gradually changed course and perused the ideas connected to Barker and Osmond (e.g., Wells 2009). The “foetal origins of adult disease” rapidly gained traction and was widely adopted in the scientific community. As the hypothesis increasingly embraced the theories of evolutionary ecology, a rich vocabulary emerged. In some cases, the terminology was drawn directly from existing ecological theories, for example, on capital and income breeders (Wells 2010), which was expanded to embrace the peculiarities of humans (Wells et al. 2019). In other cases, new concepts were coined to address specific aspects of the overarching hypothesis.

It is currently accepted that phenotype formation during embryonic and fetal development advances under the influence of various “environmental factors” (environmental cues; Wells 2006), which shape development trajectories (Mustillo et al. 2003) and/or amend “fetal programming” (Godfrey and Robinson 1998). The environmental cues are integrated across the parental lineage (generational phenotypic inertia; Kuzawa and Fried 2017) and may result in a phenotype that is poorly matched with the actual environmental conditions (Predictive Adaptive Responses; Gluckman et al. 2005; Bateson et al. 2014). The inertia is linked to biological limitations associated with maternal physical size, parity, and nutritional status, which can limit/be permissive of fetal development (Gluckman and Hanson 2004). The socioeconomic setting is often seen as critical for pregnancy outcomes because several key contributors to fetal health have associations with the mother's socioeconomic status (Wells 2023). There seems to be a convergence in the theoretical framework across multiple disciplines (e.g., Del Giudice 2014; Wells et al. 2019), and with a broader scope, we now refer to the “developmental origins of health and disease” (Hollstein et al. 2020; Wells 2023). The more comprehensive application has emphasized the benefit and cost of adaptation, which increasingly considers life‐history trade‐offs. Pathologies are often cast as biologically meaningful events, even though they negatively influence the health and well‐being of individuals (e.g., Del Giudice 2014).

Despite these advances, many unanswered questions limit the hypothesis's applicability as a model of phenotype formation in humans. What environmental cues are relevant, and how does phenotypic inertia modulate their impact? Is it meaningful to refer to development trajectories without defining a target, as described for physical growth and attainment of adult height (Tanner 1986)? Will the answers to such questions help us identify newborns who will meet health challenges in adulthood more accurately?

These questions seem particularly relevant when birth weights (BW) are used as indices of risk of elevated BMI and diabetes. “Low birth weight” (< 2500 g) was initially used as a biometric risk marker, while later studies often use other metrics such as “small‐for‐gestational‐age” (SGA) and skinfold thickness to estimate IUGR (Wilcox 2001). Contributions from maternal, fetal, placental, and environmental causes seem equally important (Suhag and Berghella 2013), and several studies suggest that differences in gestation length (GA, prematurity) also is associated with added risk (de Mendonça et al. 2020). The lack of specificity is compounded by evidence suggesting that large neonates (> 4000 g) often have a higher risk of elevated BMI than normal and low‐weight neonates (Zhao et al. 2012) and that fetal growth restriction in many cases has limited bearing on future development trajectories. Thus, twins and triplets have little to no added risk, although they typically have reduced BW (Petersen et al. 2011; Bjerregaard‐Andersen et al. 2013). Nor does seasonal variation in BW (Chodick et al. 2009) and co‐occurring differences in maternal caloric intake (Westerterp‐Plantenga 1999) lead to a clear association with adult diseases like diabetes (Jensen et al. 2015; Vaiserman et al. 2009). The meager significance of permanent (twins) and gradually developing (seasonal) nutritional deficits compare well to that observed in adults when they experience food deficits for several months (Keys et al. 1950; Prentice et al. 1992; Dulloo 2021). As suboptimal food intake continues, they gradually become leaner as they lose weight. Months later, when they eat ad libitum, they initially overcompensate and achieve a higher weight and BMI than before starvation (Dulloo 2021). However, their weight will gradually be normalized to pre‐starvation levels without any measurable physiological or metabolic change. It is, however, possible to alter physiology/physiological targets (BMI) in human adults (Kroke et al. 2002; Zou et al. 2021) and experimental animals (Brownell et al. 1986) by exposing them to recurrent weight loss and weight regain, which suggests that metabolic change can evolve through repeated stimulation of metabolic pathways. The current evidence supports that weight‐cycling may result in higher BMI. It also seems that there may be a risk of further development of diabetes (Mackie et al. 2017), but that it depends on the anatomical‐physiological reference point and the age when weight‐cycling begins, ethnicity, and so forth. (Field et al. 2004; Wells 2012; Montani et al. 2015; Yokomichi et al. 2017; Andréasson et al. 2022).

Accepting that repeated stimulation of metabolic pathways in early life (rather than an accumulated effect measured at parturition) influences development trajectories is a simple way of addressing the poorly defined association between BW and development trajectories. However, assessing the frequency of intrauterine nutrition deficiencies induced by socioeconomic stressors and infections is problematic because these phenomena would not show predictable frequencies throughout a pregnancy. Albeit, we can investigate whether the frequency of nutrient deficits is relevant because elevated temperatures negatively affect pregnancies (Roberts 1969; Wells and Cole 2002; Jensen and Sørensen 2013). Placental vasoconstriction during daily maximum temperatures can diminish nutrient delivery (Hansen 2009; Choudhari 2022) and deliver predictable high‐frequency intrauterine nutritional perturbations through diminished placental blood flow. Hence, fetal growth rates and BW should be affected in populations that live in locations with high daily temperature amplitudes. Regrettably, this interpretation seems remote from any ecological context, and it is difficult to connect to an ecological or evolutionary logic. We cannot offer simple, intuitive explanations such as those presented in earlier works (Neel 1962; Neel 1999), which, with support from WWII famine studies, proposed that historical famines occurring throughout human history favored a thrifty genotype/phenotype (Bleker et al. 2021; De Rooij et al. 2022; Tolkunova et al. 2023). Suppose we seek a past evolutionary context, which could allow humans to adapt to such perturbations or adopt daily temperature amplitudes as an environmental cue. In that case, we must look further back into our history.

Daily temperature amplitudes are an environmental index that indirectly informs of the lack of environmental resources and/or the need for an extensive home range. High daily amplitudes (up to 20°C) are observed at high altitudes and in arid regions where water bodies and vegetation are scarce or absent (Wang et al. 2014; Jang et al. 2022; see global map in Wikipedia 2024). Where rainfall, water bodies, and vegetation are abundant, daily amplitudes decline towards a minimum of five degrees Celsius. It can thus be speculated that a thrifty (oligotroph) phenotype/genotype emerged within human populations because our species lived in arid African environments for an extended period. Possibly, early humans began to cope with the energetic challenges of arid environments like other animals do (Grimaldi et al. 2015; Rocha et al. 2021). As humans later spread across the world, differences between ethnic groups emerged (Wells 2012) due to founder effects and genetic drift. Whether this elaborate vision is true or not, we must return to more recent socioeconomic “progress” to explain why the associated health challenges emerged in the 20th century (see discussion for further details).

Contemplating the impact of daily temperature amplitudes is unconventional, but at the same time, a logical extension of previous work on human plasticity in relation to climate (Roberts 1978). It has been argued that climates, that is, the long‐term effect of temperatures, influence human anthropometrics and that the response aligns with Bergman's rule (Bergmann 1847; Foster and Collard 2013; Bindon and Baker 1997; but see Bogin et al. 2022; Pomeroy et al. 2021), that is, that animals tend to attain larger size in cooler climates. As BW is associated with height (Kramer 1987), we can expect that BW is related to mean temperatures, as noted by Roberts (1969), (but see Wells and Cole 2002; Jensen and Sørensen 2013). Similar notions are given by Allen's rule (Allen 1877), which states that the body's extremities will be shortened in cooler climates. Human populations adhere to Allen's rule due to postnatal temperature growth restrictions induced by vasoconstriction (Serrat et al. 2008; Betti et al. 2015). Albeit, Allen's rule only affects populations at very high latitudes, which means that people tend to grow larger and heavier (higher BMI) with increasing latitudes (Roberts 1953; OWD 2019; GHO 2020). Last, there is a theoretical foundation for assuming that the timing of births occurs in tune with seasonal resource availability (Varpe 2017). Hence, the seasonal variation in BWs (Chodick et al. 2009) could result from seasonal physiological variations combined with a long‐term adaptation to local weather patterns (Pereira et al. 2012).

We assessed the association between annual mean temperature, seasonal temperature amplitudes, daily temperature amplitude, BW, gestational age (GA), and crude fetal growth rate (CFGR = BW/GA) in a globally distributed sample that encompassed significant differences in daily temperature variation (*n* = 163). In aggregating these for each nation (*n* = 70), we also assessed whether the global variation in calorie intake, BMI, and height is associated with average daily temperature variation. By optimizing our selection of data records (excluding data on individuals and communities) and aggregating these by country, we improve our ability to detect general patterns of adaptation. This also meant that the influence of socioeconomic and cultural factors (Anekwe et al. 2020) was suppressed, and hence the outcome primarily addresses general associations shared by larger aggregates/populations.

## 2 Materials and Methods

We analyzed and compared the outcome for several attributes, including BW, GA, CFGR, calorie intake, BMI, and height. We expected an impact of daily temperatures on BW, CFGR, and BMI. Secondary effects were likely for GA and height, while no effect was expected for calorie intake. The negative expectation was rooted in the theoretical consideration that when mammals in arid environments seek to improve their energy balance, they cannot do so by simply enlarging their home range/increasing their food intake. Adaptations in arid environments should instead involve improvements in nutrient absorption and post‐absorption processes, which also allow an increase in the production/supply of metabolic water (Rocha et al. 2021). These considerations can also explain why human anthropometric measurements in African countries indicate a higher food intake (uptake) than the food availabilities suggested by FAO's statistical records (van Wesenbeeck et al. 2009).

We compiled paired records of BW and GA, which permitted an assessment of CFGR. The dataset included geographically less accurately defined normative studies that included data from national registries from larger countries like the USA and Canada. The total sample included 163 observations from 120 locations. The “location” of national and regional samples was defined as the capital of the country, the provincial capital, or the largest city in the region. The mean year of the sample was 2000, sd 16.

We retrieved matching information on temperatures for each of the locations. Thirty‐year mean daily maximum and minimum temperatures from the warmest and the coolest months were identified for each location. We first searched for the temperatures in the online record of the World Meteorological Organization (WMO 2021). If unavailable, we searched in World Weather Online (WWO 2021), and in a single instance, we found the information on Time and Date (TAD 2021). Eighty‐three percent of the records (83%; 136/163) originated from WMO, 16% (26/163) from WWO, and 0.5% (1/163) from TAD. We used the mean temperature (mean of the four measures), seasonal temperature amplitude (the mean temperature in the warmest month minus the mean temperature in the coolest month), and the mean daily temperature amplitude from the coolest and the warmest months for analysis.

While deleting countries/regions that could not be included in the following analysis (The Faroese islands, Taiwan, etc.), the data was aggregated into 70 countries with 2.0 sd 1.7 observations per country. Means for national BW, GA CFGR, and temperatures were calculated and supplemented with national records for other population attributes. Last, we inspected whether data aggregation affected the association between temperatures and CFGR. We did this by perusing the association between CFGR and mean annual temperatures, seasonal amplitudes, and mean daily amplitudes for both data sets (*n* = 163 and 70, respectively).

### 2.1 Statistical Evaluation

The initial model for analyzing global variation in CFGR included the three mentioned temperatures, representing thermic‐environment characteristics. The initial model was:

CFGR=a×mean+b×seasonal amplitude+c×daily amplitude+Error

We then added additional variables, expecting that these would show a more specific association, and removed those variables that became non‐significant (forward selection, *p* > 0.05). We individually introduced the variables into the model to minimize the risk of identifying spurious associations due to confounding/strong cross‐correlations (Table 1). The attributes were added in the following order: (1) Calorie intake day^−1^ person^−1^ as provided by FAO (FAO 2001; FAO 2008; FAO 2020), (2) BMI (mean of male and female BMI for +18 years, GHO 2020), (3) Height (mean of male and female height, cm; OWD 2019), and (4) Economic status given as low income, lower middle income, upper middle income, or high‐income country (Gapminder 2018). When the analytical sequence was completed, all variables removed in a given analytical step were added again and removed if found non‐significant. The FAO file (FAO 2020) that provided information on calorie intake included data for 1991, 1996, 2001, and 2007, that is, covering 10 years before and after the mean year of observation. The data for other attributes included the same years.

**TABLE 1:** Pearson Correlation Coefficients for selected variables used in the statistical analyses (*n* = 70). Crude fetal growth rate (CFGR, g/day), Height (cm) BMI (weight/height^2^), annual mean temperature (°C), Seasonal temperature amplitude (°C), daily temperature amplitude in the coolest month (°C), and daily temperature amplitude in the warmest month (°C) of the year. Temperatures are 30‐year means.

	Calories day^−1^ person^−1^ (1)	Height female (2)	Height male (3)	BMI female (4)	BMI male (5)	Annual mean temperature (6)	Seasonal amplitude (7)	Daily amplitude coolest month (8)	Daily amplitude warmest month
CFGR	0.62	0.73	0.77	0.50	0.67	−0.61	0.43	−0.56	−0.29
1		0.72	0.77	0.57	0.80	−0.62	0.63	−0.49	−0.11
2			0.98	0.44	0.66	−0.70	0.61	−0.58	−0.15
3				0.50	0.74	−0.72	0.62	−0.58	−0.15
4					0.86	−0.23	0.34	−0.05	0.25
5						−0.53	0.52	−0.32	0.07
6							−0.76	0.52	0.21
7								−0.32	0.06
8									0.67

We identified the attributes associated with daily temperature amplitudes with the analytical sequence. Some associations with daily amplitudes appeared statistically weak, so we continued to add CFGR to the final model and removed the attributes that became insignificant (*p* > 0.05). We accepted a change in outcome following the addition of CFGR as an indication of “weak effects,” while an unaltered outcome implied “strong effects.” All statistical analyses were performed in SAS 9.4, SAS Institute, Cary, USA.

## 3 Results

**FIGURE 1:**
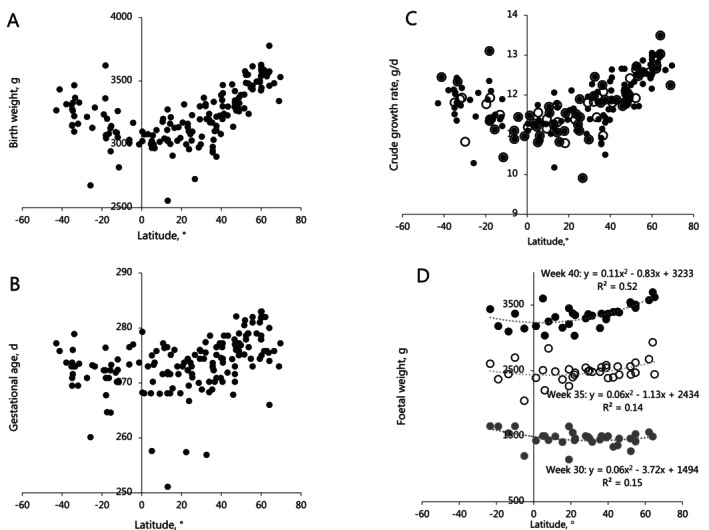
Birth weight (BW) and gestational age (GA) data overview. (A) Scatterplot of BW vs. latitude. (B) Scatterplot of GA vs. latitude (C) Crude fetal growth rates (CFGR) for individual locations vs. latitude (●, *n* = 163) and countries (○, *n* = 70), (D) Scatterplot of fetal weights for weeks 30, 35, and 40. The data on fetal weight at weeks 30, 35, and 40 was based on 86 publications on national/regional fetal growth rates aggregated to 34 nations.

Both BW and GA showed clear variation over latitudes (Figure 1A,B). CFGR showed a clear association with mean annual temperatures and daily temperature amplitudes in the non‐aggregated (Model 1, Table 2, Figure 1C) and aggregated (Model 2) data sets, indicating that aggregation did not affect the associations. The analysis of CFGR identified a significant positive association between height and BMI, while daily temperature amplitudes had a negative association (Model 3). These three variables explained about 2/3 of the global variation in CFGR.

**TABLE 2:** The statistical outcome for analyses of crude fetal growth rates ((CFGR: Birth weight (g)/gestational age (days)), Gestational age (GA) and birth weight (BW) for association with temperatures, anthropometrics, and socioeconomic status. Model 1 included 163 observations, while Models 2 to 5 included 70 observations.

Variable	Parameter	Estimate	SE	*t* value	Pr > |t|	*R* ^2^
	Intercept	13.12	0.19	68.01	< 0.0001	0.44
CFGR	Mean temperature	−0.05	0.01	−7.02	< 0.0001	
Model 1	Seasonal amplitude	0.00	0.01	−0.43	0.66	
	Daily amplitude	−0.06	0.01	−4.29	< 0.0001	
	Intercept	12.92	0.32	40.51	< 0.0001	0.48
CFGR	Mean temperature	−0.04	0.01	−3.14	0.002	
Model 2	Seasonal amplitude	0.00	0.01	0.05	0.96	
	Daily amplitude	−0.06	0.02	−2.53	0.01	
	Intercept	1.25	1.84	0.68	0.49	0.66
CFGR	Height	0.05	0.01	4.09	0.0001	
Model 3	BMI	0.10	0.03	3.46	0.0009	
	Daily amplitude	−0.06	0.02	−3.38	0.0012	
GA	Intercept	277.63	2.70	102.76	< 0.0001	0.34
Model 4	Mean temperature	−0.20	0.09	−2.23	0.03	
	Seasonal amplitude	−0.19	0.08	−2.51	0.01	
	High Income	4.01	1.79	2.25	0.02	
	Upper middle income	0.30	1.71	0.18	0.86	
	Lower middle income	−0.92	1.71	−0.54	0.59	
	Low income	0.00				
BW	Intercept	−3323.38	871.54	−3.81	0.0003	0.74
Model 5	Gestational age	13.77	3.32	4.15	< 0.0001	
	Daily amplitude	−16.44	5.06	−3.25	0.0018	
	BMI	27.25	7.92	3.44	0.001	
	Height	13.65	3.80	3.59	0.0006	

Analysis of GA yielded a relatively weak association with mean temperature and seasonal amplitudes, while socioeconomic status assigned higher GA for high‐income countries (Model 4). GA had limited global variation and was generally poorly explained (Figure 1B, Table 2, Model 4, *R* ^2^ = 0.34). Hence, the analysis of BWs led to the same outcome as the analysis of CFGR when GA was included (Model 5). Daily temperatures negatively affected BWs with 16.4 se: 5.0 g pr °C.

The analysis of calorie intake did not show any association with daily temperature amplitudes, and adding CFGR to the final model did not indicate that it was relevant in the context (*p* = 0.68, Model 6, Table 3). For BMI, Socioeconomic status explained substantial parts of the global variation, but the contribution from daily amplitudes was also quite clear (Model 7). Adding CFGR to the model did not change the outcome (Model 9). Last, the height analysis showed a clear association with mean temperatures and BMI and a weaker association with daily amplitudes and socioeconomic conditions (Model 8). Daily temperature amplitude was removed from the model after adding CFGR (Model 10).

**TABLE 3:** The statistical outcome for analyses of mean daily calorie intake, BMI, and height for association with temperatures, anthropometrics, and socioeconomic status. The models included 70 observations. The label ‘Without inclusion of CFGR’ refers to models that did not consider CFGR, while the label ‘With inclusion of CFGR” models included CFGR, and thus permit an effect of daily temperature amplitudes through CFGR.

Mode	Variable	Parameter	Estimate	SE	*t* value	Pr > |t|	*R* ^2^
Without inclusion of CFGR		Intercept	874.61	453.09	1.93	0.06	0.76
	Calories	Seasonal amplitude	12.33	4.14	2.98	0.004	
	Model 6	BMI	51.80	20.94	2.47	0.02	
		High income	775.19	149.66	5.18	< 0.0001	
		Upper middle income	471.29	140.75	3.35	0.001	
		Lower middle income	200.20	119.87	1.67	0.09	
		Low income	0.00				
		Intercept	−5.08	8.32	−0.61	0.54	0.68
	BMI	Daily amplitude	0.27	0.06	4.39	< 0.0001	
	Model 7	Calories	0.0012	0.0006	2.00	0.05	
		Height	0.13	0.05	2.42	0.02	
		High income	3.18	0.90	3.55	0.0007	
		Upper middle income	3.05	0.72	4.25	< 0.0001	
		Lower middle income	1.99	0.64	3.12	0.003	
		Low income	0.00				
		Intercept	152.78	5.11	29.93	< 0.0001	0.74
	Height	Mean temperature	−0.18	0.06	−3.23	0.002	
	Model 8	Daily amplitude	−0.36	0.15	−2.41	0.02	
		BMI	0.81	0.25	3.21	0.002	
		High income	1.91	1.95	0.98	0.33	
		Upper middle income	−0.83	1.69	−0.49	0.62	
		Lower middle income	−2.46	1.47	−1.67	0.09	
		Low income	0.00				
With inclusion of CFGR		Intercept	2.06	3.84	0.54	0.59	0.71
	BMI	CFGR	1.15	0.32	3.55	0.0007	
		Daily amplitude	0.29	0.06	4.94	< 0.0001	
	Model 9	Calories	0.0014	0.0005	2.58	0.01	
		High income	2.72	0.88	3.11	0.003	
		Upper middle income	2.44	0.71	3.46	0.001	
		Lower middle income	1.35	0.60	2.25	0.03	
		Low income	0.00				
		Intercept	127.82	8.48	15.07	< 0.0001	0.76
	Height	CFGR	3.31	0.73	4.56	< 0.0001	
	Model 10	Mean temperature	−0.12	0.05	−2.15	0.03	
		High income	3.57	1.44	2.48	0.011	
		Upper middle income	0.44	1.32	0.33	0.74	
		Lower middle income	−2.04	1.28	−1.59	0.11	
		Low income	0.00				

## 4 Discussion

The outcome of the analysis of CFGR was consistent with previous research showing that BW is strongly associated with maternal height and some measure of food intake (calories or BMI; Kramer 1987). There is, however, no direct association with socioeconomic conditions, even though they, without exception, influence adult metrics. The analysis of BMI also delivers the expected results, as it would be almost inevitable that calorie intake and socioeconomic status would explain a substantial part of the global variation in BMI (Azam and Idrees 2019). In both analyses, daily temperature amplitudes offer significant explanatory power when considering the main known contributors. The same is not the case for GA, calorie intake, or height, even though they also have associations with (other) environmental and/or socioeconomic factors.

Each of the variables in Models 3 and 7 points to a set of “causes” contributing to CFGR and BMI variation. However, viewing the associations as present or absent is not helpful because the attributes are mutually dependent. Thus, CFGR has no direct link to socioeconomic conditions, but there is an indirect association through its association with height. When we acknowledge that nutritional, energetic, and epidemiological environments contribute to differences in height (Pomeroy et al. 2021; Gowland and Walther 2018; Tanner 1990), we must accept that this is also partly true for CFGR. Our comments on the BMI analysis would be similar, but we would stress the importance of dietary components and socioeconomic conditions (Kuzawa and Eisenberg 2012). In comparing the outcomes, CFGR and BMI are coupled, such that BMI increases CFGR (Model 3) and CFGR increases BMI (Model 9), and there are no negative contributions in these simple models that would allow BMI and CFGR to stabilize. Hence, high parental BMI supports high BMI in the following generations (Gilley et al. 2023), perpetuated by a range of physiological and behavioral factors (Blair et al. 2020). The impact of daily temperature amplitude on BMI works independently of this association. We could speculate that altered sleeping patterns, daytime reductions in physical activity (Booth et al. 2012), and reduction in body temperatures during the day (Grimaldi et al. 2015) deliver added negative feedback by reducing the impact of daily temperatures for people with an elevated BMI. Unsurprisingly, reducing calorie intake and augmenting energy expenditure (e.g., Böckerman et al. 2010) will reduce BMI and CFGR (Gentil et al. 2020).

The interpretation of the analysis of height is more straightforward because we only acknowledge three contributors (Table 3, Model 10). Changes in height are typically allometric such that the extremities (tibia and femur) contribute relatively more to changes in height than changes to the upper body (Tanner 1990; Jantz and Jantz 1999), but all parts of the body will be elongated during height increments. Ad libitum intake of high‐quality food (e.g., animal protein) is known to be permissive of added juvenile growth (Grasgruber et al. 2016), but high protein intake may eventually have a negative impact (Xiong et al. 2023). Conversely, high energy expenditure (physical work) and infectious diseases limit growth (Tanner 1990; Hedges et al. 2017). Here we note that the factors that permit high CGFR also permit later growth, while low mean temperatures and poor financial settings have a negative effect. Mean temperature likely affects the growth of body extremities (Serrat et al. 2008; Betti et al. 2015), while other factors such as physical work (lower incomes) have a more generally negative impact (e.g., Böckerman et al. 2010).

The level of explanation in the models is relatively modest. This is partly due to the (forced) assumption that the attributes of the mothers (and fathers) in the BW records matched the characteristics of the general population in the calorie, BMI, and height records. If the general population did not experience temperatures similar to those of the mothers who delivered the infants, it would also be a source of error. Other significant contributions were linked to the relatively low accuracy for BWs and GAs (Skupski et al. 2017), which in equal terms would apply to different attributes like calorie intake (Cialfa et al. 1991; Thar et al. 2020). It is, however, more meaningful to emphasize that we only considered key contributors that define the “maternal phenotype” (food intake, height, weight). The lack of information on parity and nuptial status (Kramer 1987), work‐life (Spencer et al. 2022), macronutrient intake (protein, fat, carbohydrates; Lechtig et al. 1975), micronutrient supplements (iron, folic acid, etc. Christian 2010), microorganism infection (measles, influenza, malaria, etc., e.g. Siegel and Fuerst 1966), and so forth, limits our ability to explain the total global variation in BW and CFGR (*R* ^2^ < 0.75). The importance of these omissions is more evident in the analysis of GA (Table 2; Model 4; *R* ^2^ < 0.34) because the maternal phenotype (height, weight) has a weaker association with GA than with CFGR and BW (Kramer 1987; Savitz and Pastore 1999).

It could also seem disconcerting that we assign importance to relatively small differences in CFGR. The small number and even minor differences in CFGR result from dividing BW with GA (Figure 1C), that is, the average weight gain per day for the entire pregnancy. However, global differences in fetal weight are relatively slight at week 30 (Figure 1D); hence, the worldwide pattern of BW emerges in the last 5–10 weeks before parturition. With this, we would argue that the “true” differences in daily fetal weight gain are several‐fold higher, as they emerge in the last 20%–25% of the pregnancy. The significance of daily temperature amplitudes is perhaps better appreciated from the analysis of BW (Table 2, Model 5), which indicates that the daily temperature amplitude negatively affects BW with approx. 16 g per degree. Since daily temperature amplitudes vary globally between 5°C and 15°C, we may account for a 160 g difference in BW, representing a sizable part of the total global variation in BW (3000 to 3700 g, Figure 1A). Other contributions from temperatures are channeled through height and calorie intake, which account for the diverse contribution of the thermal environment to CFGR and BMI (Roberts 1953). The association seems compelling, but it is impossible to determine whether a nutritional deficit equaling 50–100 g BW will change fetal development trajectories or whether changes emerge from “falling slightly short” every day for 1–2 months. However, in logical terms, daily temperature amplitudes must deliver an impact day by day either during the pregnancy or by modulating the maternal phenotype. Arguing that daily amplitude works by altering the maternal phenotype could inspire the hypothesis that mothers to some extend protect their unborn children from daily nutritional perturbations by reducing the nutrient flow, that is, they adopt daily amplitudes as an environmental cue. To provide more compelling evidence of an associated change in fetal development trajectory in the gestational period, we must peruse the given associations for multiple GAs and show that the association changes during the pregnancy, for example, a high‐calorie intake is associated with higher fetal weight in week 40 but a lower fetal weight in week 30.

Multivariate analysis of cross‐sectional data records is a standard testing platform for initial analyses. However, it cannot carry the burden of evidence alone because pre‐analysis choices (data selection, transformations, data omissions, etc.) substantially influence the outcome. Our comparative approach may carry more weight, as we can discriminate between two attributes that should be affected by daily temperature amplitudes (CFGR and BMI) and one that should not (calorie intake). These ideas are, however, also of interest because they add cohesion across physiological and ecological aspects of the hypothesis. With the impact of daily temperature variation, we assign importance to physiological processes because a diurnal temperature range of 15°C to 25°C has the same impact as a variation from 25°C to 35°C. We must refer to acclimation and other types of adaptation processes to account for the claim that a daily variation of 10°C has the same impact on populations living under different mean temperatures. It will, however, require substantial efforts to fully establish that our thermal environment plays a crucial role in human phenotype formation, not only for the effect of daily temperatures on CFGR but also for other associations, such as, for example, the effect of (seasonal) temperatures on calorie intake (table 3, Model 6, Johnson and Kark 1947).

**FIGURE 2:**
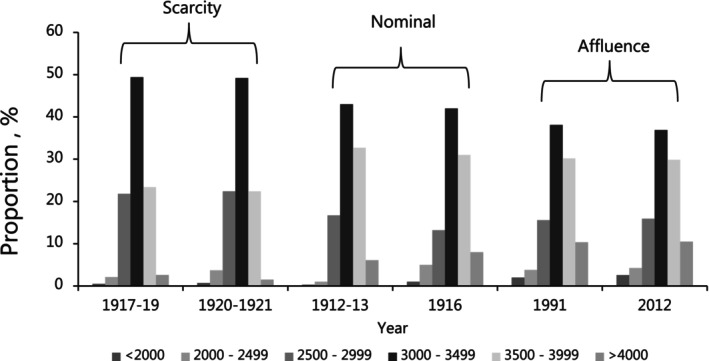
Changes in birth weights in Germany in the 20th and 21st centuries. Birth weight distributions in Germany as defined by daily calorie intake per person day^−1^. “Scarcity” refers to calorie intake of less than 2000 cal and mean birth weights of approx. 3100 g, “Nominal” refers to 2150 to 2850 cal and a mean birth weight of approx. 3250 g. “Affluence” refers to approx. 3350 cal, which matches a mean birth weight of 3350 g. Germany entered the epidemiological transition stage 2 in 1930–1934.

The next logical step would be to determine whether daily temperature amplitudes are associated with patterns of infectious disease mortality because this would allow for a more comprehensive understanding of the environmental factors related to thriftier phenotypes (Zoppini et al. 2018; Ragsdale et al. 2019; Corona et al. 2021). As a first step, we would connect the “developmental origins of health and disease hypothesis” with the epidemiological transition theory (Omran 1969; Santosa et al. 2014), as it allows us to appreciate how the impact of historical famines, and negative impact on pregnancies, varied with socioeconomic “progress”. Initial studies on the famine in the Netherlands (1945/1946) showed that a reduction in BW of approx. 200 g was associated with various adverse health impacts and documented changes in the life history of individuals exposed to the famine (Bleker et al. 2021; De Rooij et al. 2022). Less persuasive findings have, however, been reported from other war‐induced famines in for example, Japan (Matsushima et al. 2018) and Korea (Han and Hong 2019); and recent studies have only shown a limited impact of famines in Russia (Tolkunova et al. 2023) and Ethiopia (Arage et al. 2020). Despite their differences, these observations are not ambiguous but underline that an inspection of later life developments requires a high survival rate among low BW neonates during the famine (Lindeboom and Van Ewijk 2015). In the terminology of epidemiological transition theory, we would state that the later life impacts of famines become apparent late in Stage 2 (“the age of receding pandemics,” that is, after infant and child mortality is reduced), which the Netherlands entered in 1910–1914 (Santosa et al. 2014). Other European countries, like Italy, were soon to follow, and it is here we find that the decrease in perinatal mortality was associated with a divergence in BWs (Ulizzi and Terrenato 1992). The same is evident in Germany, where the records also allow for an assessment of calorie intake, mean birth weight, and the BW distribution over a century (Figure 2), which can be interpreted in terms of scarcity and affluence. Ninety‐two (92%) of German mothers delivered neonates with BW of 2500 to 3999 g in 1912 (Peller and Bass 1924, Figure 2), which was reduced to 82% in 2012 (UN 2023). The numbers for the neighboring country, Denmark, were 85% in 1968 and 80% in 2008 (UN 2023), which could explain the increase in obesity and diabetes in these countries (e.g., Thomsen et al. 1999). We have not implicitly considered the epidemiological stage in our analyses because it is of little relevance in the study of the mean BW. We also believe that it is a fair assumption to accept income groups as proxies for the epidemiological stage and, hence, that appropriate adjustments have been made in the analysis of BMI and height. High income could thus be understood as Stage 4, under which a higher proportion of low and high BW neonates are born and survive to adulthood. Even though our understanding of BW diversification under the epidemiological transition remains unclear, we accept that health challenges in current populations emerged under a weakening stabilizing selection (Ulizzi and Terrenato 1992). The scale of the phenomena encompassed by the “fetal origin of adult diseases hypothesis” is thus likely contingent on the BW divergence associated with socioeconomic “progress”.

## 5 Conclusion

Our simple assessment considered that thriftier phenotypes are associated with high‐frequency temperature variations, which we explored by parallel analysis of global variation in CFGR and BMI. We showed that daily temperature amplitudes add to the level of statistical explanation for both CFGR and BMI but not for other population attributes. It must, however, be emphasized that the association between BW and BMI/obesity/diabetes is contingent on the epidemiological environment because it unfolds under low perinatal, infant, and child mortality rates. We also find that the models are mutually consistent and that the outcome “tally up” in a statistical sense, which supports ideas of interdependency across anthropometrics and traits, as it is accepted under the developmental origins of health and disease hypothesis.

## Author Contributions

Per M. Jensen: conceptualization, data curation, formal analysis, methodology, visualization, writing – original draft. Marten Sørensen: visualization, writing – review and editing.

## Ethics Statement

The authors have nothing to report.

## Conflicts of Interest

The authors declare no conflicts of interest.

## Data Availability

The authors have nothing to report.
